# Diverse Roles of Tubulin Polymerization Promoting Protein 3 (TPPP3) in Human Health and Disease

**DOI:** 10.3390/cells14201573

**Published:** 2025-10-10

**Authors:** James W. Lord, Sachi Horibata

**Affiliations:** 1 Precision Health Program, Michigan State University, East Lansing, MI 48824, USA; lordjame@msu.edu; 2 Cell and Molecular Biology Program, Michigan State University, East Lansing, MI 48824, USA; 3 Department of Pharmacology and Toxicology, College of Human Medicine, Michigan State University, East Lansing, MI 48824, USA

**Keywords:** tubulin, TPPP3, microtubules, microtubule-associated proteins (MAPs)

## Abstract

The tubulin polymerization promoting proteins (TPPPs) are a small family of conserved proteins originally characterized as microtubule binding proteins. TPPP1, the first identified member, both binds to and bundles microtubules. Its homologs, TPPP2 and TPPP3, are encoded by separate genes on distinct chromosomes but both lack the N-terminal tail present in TPPP1. Functional studies revealed that TPPP3 retains comparable microtubule binding and bundling capacity to TPPP1, whereas TPPP2 displays markedly reduced binding and no bundling activity. Intriguingly, TPPP3 has been implicated in many different diseases. In this review, we summarize the current findings on TPPP3 and its dysregulation in various diseases including cancer, reproductive dysfunction, musculoskeletal conditions, endothelial dysfunction, and neurodegenerative diseases.

## 1. Introduction

**Figure 1:**
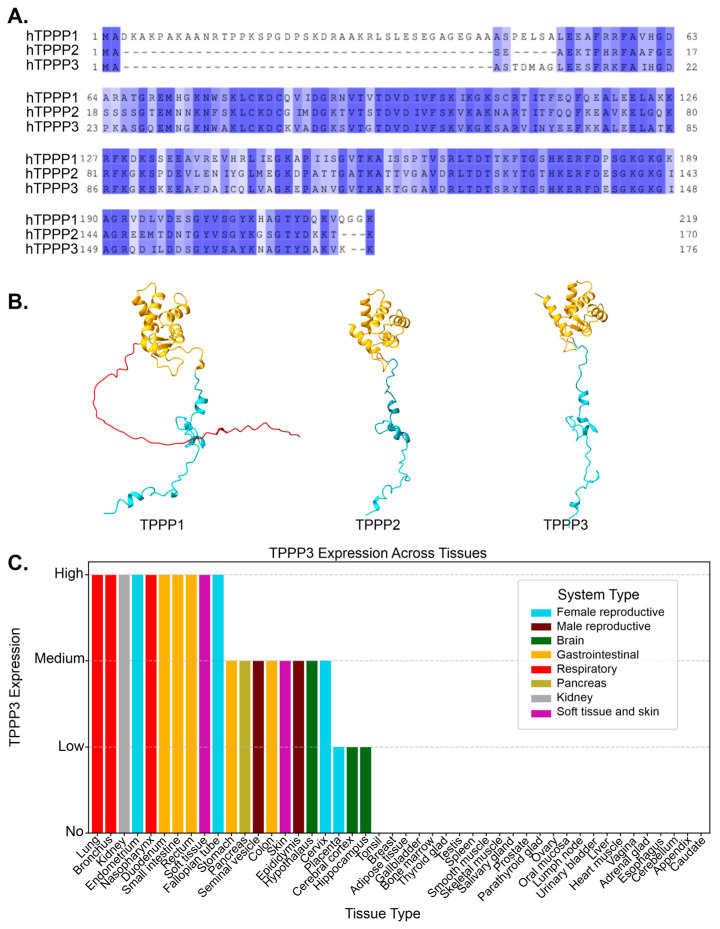
(**A**) Alignment of protein sequences between TPPP1, TPPP2, and TPPP3. Residues are shaded by conservation. Dark blue shading indicates highly conserved residues. The degree of conservation decreases with lighter shades, and white indicates non-conserved regions. (**B**) AlphaFold-predicted structures of TPPP1, TPPP2, and TPPP3, highlighting structural similarities among the three proteins. TPPP2 and TPPP3 lack the N-terminal region present in TPPP1. Red = N-terminus, Blue = C-terminus, Yellow = Ordered helical domain. (**C**) TPPP3 expression level in different tissues, taken from the proteinatlas.org. Each tissue type is composed of multiple different cell types.

Tubulin polymerization promoting proteins (TPPPs) are a family of three microtubule associated proteins (MAPs) that were initially characterized as microtubule binding proteins [1]. TPPP1, also referred to as TPPP/p25, was the first of these proteins to be isolated from bovine brain extracts and was originally thought of as a brain-specific protein, as it was also found to localize in oligodendrocytes [2,3]. In oligodendrocytes, TPPP1 was able to nucleate microtubules from the golgi stacks [4]. TPPP1 also bound to and promoted the assembly of microtubules [5]. Two other members of the TPPP family, TPPP2 and TPPP3, were later identified due to their shared homology encoded by separate genes on distinct chromosomes [1]. Functional analysis of the three proteins revealed that TPPP3 and TPPP1 bind, stabilize, and bundle microtubules, while TPPP2 is only able to weakly bind tubulin and did not have any bundling capacity [1]. At the amino acid level, TPPP1 and TPPP3 share 81% sequence similarity excluding the N-terminal region, as TPPP3 does not have the intrinsically disordered N-terminal region (Figure 1A,B) [6]. While TPPP2 and TPPP3 share a substantial 76% sequence similarity, differences in their sequences has resulted in reduced microtubule-binding affinity and an inability to promote microtubule bundling [1].

TPPP1 is the most extensively studied member of the TPPP family, with numerous *in vitro* [5,7,8] and *in vivo* studies [4,9,10], multiple knockout models in various organisms including *Drosophila* and mice [4,9], and strong evidence linking it to the development of neurological disorders [11,12]. Given TPPP3’s sequence similarity to TPPP1 and its shared ability to bundle microtubules, its normal biological functions have been sparsely studied. However, over the past nearly two decades, TPPP3 has been found to be implicated in a number of different diseases, with minimal microtubule association and acting on seemingly unrelated cellular pathways. TPPP3 is not expressed in most cell types, but is widely found in different tissues including the brain, gastrointestinal tract, airway, and reproductive tract (www.proteinatlas.org) (Figure 1C) [13]. In this review, we have compiled findings related to TPPP3 and how its dysregulation is involved in various diseases.

## 2. Cancers

### 2.1. Cervical Cancer

TPPP3 was first implicated in cancer shortly after its initial discovery. Zhou et al. sought to investigate endogenous functions of TPPP3 in the HeLa cervical cancer cell line. Using RNA interference (RNAi), they assessed the impact of TPPP3 knockdown on cell proliferation and apoptosis [14]. Loss of TPPP3 expression resulted in an increase in cells in the G2-M phase of the cell cycle and a decrease of cells in S phase, indicating that more cells are becoming stalled at the G2 checkpoint and fewer cells are entering the cell cycle. Loss of TPPP3 also leads to an increase in apoptotic cells. Cells with depleted TPPP3 were found to have a varying number of mitotic spindle poles, most with four or five. Multipolar cells can lead to aneuploidy which can ultimately lead to cell death [15,16]. They later expanded on this initial study in Lewis lung carcinoma, finding that TPPP3 depletion also inhibited tumor growth as well as metastasis [17].

### 2.2. Non-Small Cell Lung Carcinoma

**Figure 2:**
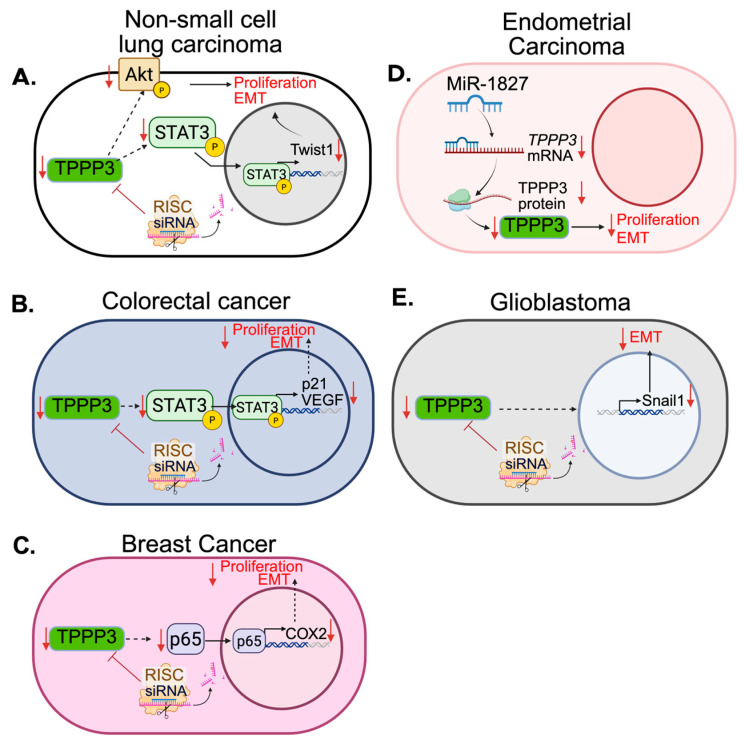
Illustrations of the described mechanism of TPPP3 in non-small cell lung carcinoma, endometrial carcinoma, colorectal cancer, glioblastoma and breast cancer. (**A**) Knockdown of TPPP3 decreases phosphorylation of Akt and STAT3 leading a decrease in proliferation and EMT. (**B**) Knockdown of TPPP3 in colorectal cancer decreases phosphorylation of STAT3, a decrease in p21 and VEGF expression and decreased proliferation and EMT. (**C**) Knockdown of TPPP3 in breast cancer decreased nuclear translocation of p65, decreased COX2 expression and decreased proliferation and EMT. (**D**) MiR-1827 can target TPPP3 mRNA decrease protein expression and leading to a decrease in proliferation and EMT. (**E**) Knockdown of TPPP3 in glioblastoma lead to a decrease in Snail1 expression and a decrease in EMT.

Furthermore, in 2016, the same group investigated the role of TPPP3 in non-small-cell lung carcinoma [18]. Looking at patient survival data on non-small cell lung carcinoma showed that patients with high TPPP3 expression had worse overall survival. *In vitro* and *in vivo* experiments showed that loss of TPPP3 expression significantly decreased cell proliferation and tumor growth. In addition, TPPP3 knockdown also induced cell apoptosis and cell cycle arrest. Phosphorylation of AKT and STAT3, proteins involved in proliferation and apoptotic signaling, decreased after knockdown of TPPP3 [19,20,21]. Consistent with this, in 2018 Li et al. also reported that TPPP3 promotes proliferation and invasion in non-small-cell lung carcinoma via the STAT3/TWIST1 pathway, providing further evidence to the potential role of TPPP3 in these cancers (Figure 2A) [22]. Overexpression of TPPP3 in non-small-cell lung carcinoma cell lines promoted cell proliferation as well as migration and invasion. There was an upregulation of transcription factors such as c-Myc and Twist1 as well as an increase in phosphorylation of STAT3. Another study from 2015 reported that cytoplasmic p27, also known as cyclin dependent kinase inhibitor 1B (CDKN1B), can promote epithelial to mesenchymal transition (EMT) and metastasis via the STAT3-TWIST1 pathway in human mammary epithelial cells, breast cancer, and bladder cancer [23]. Phosphorylation of p27 is linked to PI3K/AKT and STAT3 activation whereby STAT3 induces Twist1, which promotes EMT. However, there is no current data linking p27 and AKT to TPPP3.

### 2.3. Colorectal Cancer

In 2017, it was reported that TPPP3 plays a similar role in colorectal cancer [24]. In this study, analysis of 96 patient samples compared to patient-matched healthy tissue revealed that TPPP3 was significantly increased in colorectal cancer. Additionally, TPPP3 protein and mRNA levels were measured in five colorectal cancer cell lines compared to normal tissue and were significantly upregulated. High TPPP3 expression was also correlated with a worse overall survival in the 96 patients. The shRNA-mediated knockdown of TPPP3 decreased cell proliferation *in vitro* and also decreased migration and invasion in colorectal cancer cell lines, LOVO and SW620. In addition, phosphorylation of STAT3 decreased after knockdown and expression of the anti-apoptotic protein BCL-2 decreased, while the expression of the pro-apoptotic protein, Bax, increased, which aligns with their previous research in non-small-cell lung carcinoma. Cell cycle analysis revealed a decrease of S phase cells after TPPP3 knockdown accompanied by a downregulation of VEGF expression. When looking at the effect of TPPP3 knockdown on angiogenesis, they found that in HUVEC cells, loss of TPPP3 resulted in fewer tube structures when these cells were treated with colorectal cancer cell-conditioned media. Protein analysis also revealed that the expression of VEGF, a pro-angiogenesis factor, was decreased in the knockdown cell lines. The mechanism is illustrated in Figure 2B.

### 2.4. Breast Cancer

In breast cancer, TPPP3 is also involved in cell proliferation, invasion, and metastasis. However, its involvement is in the context of NF-κB/COX2 signaling [25]. In breast cancer, Nuclear factor kappa-light-chain-enhancer of activated B cells (NF-κB) signaling is aberrantly activated and contributes to chemoresistance and metastasis [26,27]. Cyclooxygenase 2 (COX-2) is also upregulated in many cancers, including breast cancer, and its expression can induce NF-κB signaling [28,29]. Silencing of TPPP3 in breast cancer cell lines MCF-7 and T47D decreased cell proliferation, invasion and migration [25]. Matrix metalloproteinases MMP-2 and MMP-9 were also found to be significantly reduced after TPPP3 silencing. Both of these proteins are frequently upregulated in cancers and are associated with metastasis and tumor aggressiveness [30]. NF-κB p65, also known as RelA, and COX2 expressions were also significantly downregulated after TPPP3 silencing, indicating that interfering with TPPP3 alters the malignant phenotype of breast cancer by downregulating the NF-κB/COX2 pathways (Figure 2C). Additionally, TPPP3 has also been shown to be associated with breast cancer lung metastasis through Tppp3^+^ monocytes [31]. Single cell RNA sequencing of breast cancer lung metastases in mice revealed a subpopulation of Tppp3^+^ monocytes that are highly associated with the metastases, likely through the promotion of angiogenesis [31].

### 2.5. Endometrial Cancer

Similarly, in 2021, Shen et al. found that suppressing TPPP3 expression in endometrial cancer also inhibited proliferation, invasion and migration [32]. When compared to normal endometrial tissue, endometrial cancer cell lines, as well as patient samples, exhibited elevated levels of TPPP3. ShRNA knockdown of TPPP3 attenuated proliferation, invasion, and migration potential, as seen in breast cancer [33], colorectal cancer [24], and non-small-cell lung carcinoma [18,22]. In addition to proliferation and metastasis, they also investigated how overexpression of microRNA 1827 (miR-1827) affects TPPP3 expression in endometrial cancer. MiRNAs have been studied for decades for their oncogenic/tumor suppressive role in cancer and potential therapeutic targets [34,35,36]. Here, miR-1827 can target the 3’-UTR of TPPP3 mRNA leading to its degradation (Figure 2D). Conversely, overexpression of TPPP3 in endometrial cancer cells with induced miR-1827 expression is able to maintain their proliferative, invasive, and migrative potential. Expression of MMP-2 and MMP-9 are also significantly decreased after loss of TPPP3 expression via miR-1827 sequestration, suppressing tumorigenesis [32]. Similar results were also seen in acute lymphoblastic leukemia where circular RNA circMUC16 targets miR-1182 which also targets the 3’ UTR of TPPP3 [37]. Silencing of circMUC16 increased miR-1182 and decreased TPPP3 expression, exhibiting anti-tumor effects.

### 2.6. Glioblastoma

TPPP3 has also been associated with stemness and EMT in glioblastoma. In 2019, a study to characterize rare genes in glioblastoma found TPPP3 contributed to worse overall survival and was associated with increased invasiveness [38]. It was later discovered that TPPP3 contributes to EMT via controlling the regulation of Snail family transcriptional repressor 1(SNAI1), a transcription factor that regulates the repression of E-cadherin, an adhesion molecule downregulated during EMT (Figure 2E) [39]. In glioblastoma, TPPP3 expression is elevated as compared to normal tissue, and its expression appears to increase with the grade of the tumor. Grade IV gliomas showed significantly higher levels of TPPP3 compared to grade I, and even more so to normal brain tissue. Glioblastoma cell lines that overexpressed TPPP3 showed decreased levels of epithelial marker E-Cadherin and increased levels of mesenchymal markers, N-cadherin and Vimentin. They also demonstrated an increased capacity for invasion and migration. Importantly, knockdown of TPPP3 led to a decrease in Snail1 expression and overexpression of Snail1 in TPPP3-knockdown cells rescued its invasive and migrative potential. Snail1/2, ZEB1/2 and Twist1/2 are all core transcription factors that can activate the EMT program [40]. Interestingly, in this study, Twist expression is not affected by TPPP3 knockdown like what was seen in non-small-cell lung carcinoma [22]. TPPP3 appears to be able to regulate EMT in multiple different cancers, but through different mechanisms. Snail transcription factors are regulated by GSK3β while Twist transcription factors are regulated by MAPK [40]. There is evidence that these transcription factors can regulate each other, i.e., Snail upregulating Twist [41]; however, it still remains unclear how TPPP3, a microtubule bundling protein, is involved in the expression of these transcription factors.

### 2.7. Liver Cancer

In 2024, Li et al. investigated how fluid shear stress, a mechanical force from blood circulation, impacts the metastatic potential of hepatocellular carcinoma [42]. Fluid shear stress is one of the hurdles cancer cells have to overcome in order to successfully metastasize. They exposed hepatocellular carcinoma cell line HepG2 to fluidic shear stress for one, two and three hours in a microfluidics chamber and then analyzed changes in gene expression using RNA sequencing. A key finding was the significant upregulation of TPPP3 in cells subject to fluidic shear stress, with the three-hour exposure group showing an approximate 80-fold increase in TPPP3 mRNA. Further experiments revealed that overexpression of TPPP3 in HepG2 cells substantially increased their viability and metastatic potential in both *in vitro* and *in vivo* models [42]. Conversely, knockdown of TPPP3 led to decreased colony formation and cell viability. Clinical data support these findings, demonstrating that high TPPP3 expression in hepatocellular carcinoma patients correlates with worse overall survival [42].

### 2.8. Pancreatic Cancer

Thus far, we have discussed how TPPP3 has a pro-tumorigenic role in multiple different cancer types. However, in 2018, a research group sought to identify potential prognostic biomarkers in pancreatic ductal adenocarcinoma [43]. They found that high expression of TPPP3 was found only in patients they deemed long survivors. Low TPPP3 was associated with significantly worse overall survival.

### 2.9. Nasopharyngeal Carcinoma

In 2020, Yang et al. carried out a pan cancer analysis of the role of TPPP3 in different cancer types [44]. They found that in head and neck squamous carcinomas, specifically nasopharyngeal carcinoma, TPPP3 expression was significantly lower than that of normal tissue. Patient survival data also indicated that a low level of TPPP3 is associated with poor prognosis for nasopharyngeal carcinoma. In addition, they found that TPPP3 expression also correlated with levels of immune cell infiltration in the tumor. There was a negative correlation between CD8+ T cells and memory B cells, but a positive correlation to levels of dendritic cells. The authors hypothesized TPPP3 has a role in the regulation of immune cell infiltration in head and neck squamous cell carcinomas. In 2022, Xiao et al. found that in oral cell squamous cell carcinoma, the most common type of head and neck cancer, higher TPPP3 expression was protective and was correlated with better overall survival, and was also associated with immune cell infiltration [45]. This is further evidenced by a publication in 2024 that identified TPPP3, as well as Mucin4 (MUC4) and Chloride intracellular channel 6 (CLIC6), as three genes involved in the post-translational modification known as lactylation [46]. Lactylation, the process of adding lactate groups to lysine residues, has been shown to modulate immune responses and metabolic adaptation as well as contribute to cell proliferation, and in nasopharyngeal carcinoma may contribute to immune evasion and tumor aggressiveness [47]. Similarly to what was found previously, all three of these genes, including TPPP3, were all downregulated in nasopharyngeal carcinoma and associated with worse survival rates.

TPPP3 appears to have differing roles in different cancers (Table 1). On one hand, it promotes tumorigenesis and metastasis in various cancers through similar pathways; on the other hand, it appears to play a protective role in others. This indicates that TPPP3 plays vastly different roles in different cell types and likely has other functions beyond microtubule polymerization and bundling. From modulation of STAT and AKT signaling, Snail and Twist signaling, NF-kB signaling, immune cell infiltration, and metastases promotion through TPPP3+ immune cells, further studies will need to be conducted to fully elucidate the role of TPPP3 across diverse biological and disease contexts.

## 3. Reproduction

Two major phases of early pregnancy, blastocyst implantation and decidualization of the uterine lining, are crucial for establishing a viable pregnancy [48]. These processes require an intricate network of signaling molecules and tight genetic control [49,50]. Following ovulation, the endometrium undergoes significant morphological changes, ultimately developing into the secretory endometrium, a stage at which it becomes receptive for embryo implantation [49,50]. Given how tightly regulated these processes are, disruption to any of these steps can lead to pregnancy failure [51].

In 2014, Manohar et al. conducted a large proteomic analysis of uterine tissues from the early-secretory and mid-secretory phase from multiple different women with unexplained infertility, and compared the proteome to that of fertile women [48]. TPPP3 expression was significantly decreased during the mid-secretory phase in infertile women. However, the exact role of TPPP3 during this phase was not elucidated during this study.

**Figure 3:**
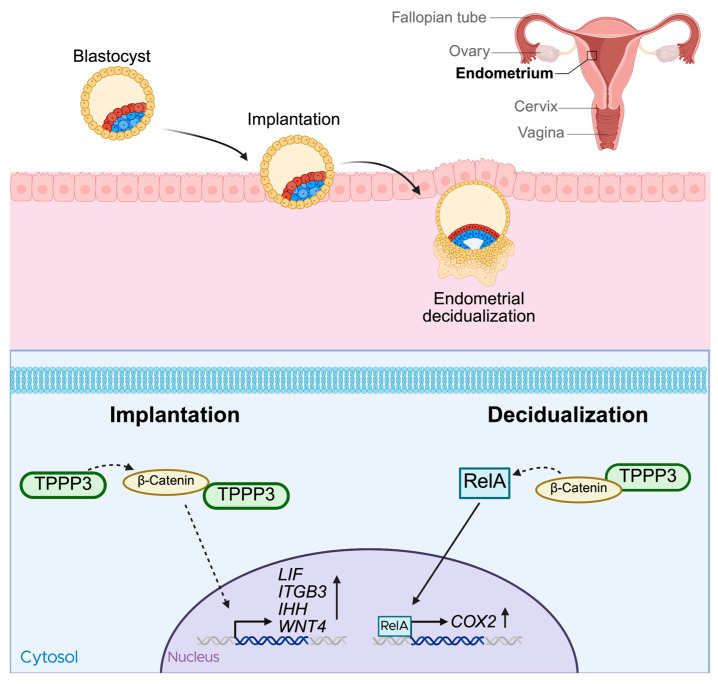
Role of TPPP3 in implantation and decidualization. Blastocyst implantation into the endometrium followed by decidualization are two crucial steps during early pregnancy. β-Catenin is a crucial protein in early pregnancy and successful implantation is marked by an increase in receptivity markers, LIF, ITGB3, IHH, and WNT4. Loss of TPPP3 results in decreased β-Catenin and also downregulation of the receptivity markers and ultimately failed implantation. During decidualization, loss of TPPP3 inhibits the translocation of RelA to the nucleus and prevents the transcription of COX2, which is required for successful decidualization.

Subsequent research by the same group in 2018 investigated the role of TPPP3 during embryo implantation [52]. TPPP3 protein expression steadily increased during the early-secretory phase, and into the secretory, or receptive phase. Crucially, knockdown of TPPP3 with siRNA significantly impacted the success of embryo implantation into the uterus. Known receptivity markers such as leukemia inhibitory factor (LIF), Integrin-β3, Indian hedgehog signaling molecule (IHH), and Wnt4, which are typically upregulated during embryo implantation [53,54,55,56], were all found to be decreased after TPPP3 knockdown, further supporting the importance of TPPP3 on implantation. The study also revealed that TPPP3 regulates β-catenin signaling and is a direct binding partner of β-catenin. Wnt/β-catenin signaling is a crucial pathway during early pregnancy [57,58] (Figure 3).

Another important step of early pregnancy is decidualization. Decidualization is the transformation of endometrial stromal fibroblasts into secretory cells, that provide nutrients and support for the implanted embryo [59]. TPPP3 is also highly important during this process [25]. Shukla et al. performed *in vitro* experiments using human embryonic stem cells and in vivo studies in BALB/c mice [25]. TPPP3 was highly expressed during decidualization, and siRNA-mediated knockdown significantly decreased endometrial decidualization [25]. Consistent with their previous findings, TPPP3 co-localized with β-catenin, and TPPP3 knockdown reduced both their localization and expression of β-catenin. Moreover, TPPP3 knockdown also decreased the nuclear localization and activation of NF-κB subunit p65, also known as RelA, in decidual cells. Given that COX-2 deficiency in mice has been shown to result in decidualization failure [60] and that the COX-2 promoter region contains multiple RelA binding sites [61,62], the observation that COX-2 expression was significantly decreased in TPPP3 siRNA-transfected human embryonic stem cells further highlights the role of TPPP3. In summary, the loss of TPPP3 expression during decidualization was found to result in decreased β-catenin signaling, a pathway capable of modulating inflammatory signaling via its components interacting with RelA. This reduction in β-catenin signaling led to less nuclear localization of RelA, subsequently decreasing the transcription of one of its targets, COX-2, which is essential for successful decidualization (Figure 3).

## 4. Musculoskeletal System

Interestingly, TPPP3 is also associated with the musculoskeletal system, where it serves as a marker for specific tendon stem cell populations. In 2009, Steverovsky et al. identified tendon-specific genes during various stages of embryonic development [63]. They found that TPPP3 was expressed in tissues surrounding the tendon, including the epitenon, paratenon, tendon sheath, and synovial joint cavitation sites. It was not until years later that TPPP3 was recognized as a marker of tendon stem cell populations [64]. These Tppp3^+^ populations that support tendon wound healing also coexpressed platelet-derived growth factor alpha (Pdgfra), a protein involved in tendon growth and remodeling [65]. Interestingly, Tppp3^+^Pdgfra^+^ tenocytes were shown to support tendon wound healing, whereas Tppp3^+^Pdgfra^−^ tenocytes were associated with fibrotic scarring in regeneration [64].

Similarly, Yea et al. also investigated wound healing after trauma, specifically heterotopic ossification, or the deposition of aberrant bone in muscles tendons or ligaments [66]. Heterotopic ossification is a common occurrence in traumatic conditions such as arthroplasty and fractures [67]. They found that Tppp3^+^ tendon sheath progenitor cells expanded rapidly after an Achilles injury in mice [66]. Single-cell RNA sequencing of tendon-associated traumatic heterotopic ossification found Tppp3 may be an early progenitor marker for either tendon or bone producing cells. They investigated Tppp3^+^Pdgfr^+^ and Tppp3^+^Pdgfr^−^ progenitor cell populations, and while the former was associated with trauma healing and the later associated with fibrosis, both of these populations gave rise to heterotopic ossification-associated cartilage [66]. Thus far these studies were done in mouse models; however, a similar phenotype of TPPP3^+^ chondrogenic cells was later described in human tissues as well [68].

Moreover, another study investigated underlying mechanisms of tendon regeneration in neonatal mice [69,70]. They highlighted that the tendon wound healing capabilities of young children are much better than that of adults and sought to identify the mechanism underlying this change [69,71]. Using neonatal mice, they revealed that Tppp3-expressing paratenon sheath cells are a key population of tendon-regenerating and PI3K-Akt signaling drives regeneration of injured tendons in neonatal mice. The PI3K-Akt pathway is associated with proliferation [20,72] and is more upregulated in the injury areas of neonatal mice as opposed to adult mice [70]. Additionally, suppression of PI3K-Akt signaling resulted in weakened and thinned tendons after regeneration, highlighting the role of PI3K-Akt signaling in these Tppp3^+^ paratenon sheath cells.

Similarly, another group investigated the underlying mechanisms of Dupuytren’s contracture, a fibrotic disease affecting flexion in affected fingers, impairing hand function [73]. At the site of the contracture, there is an infiltration of Tppp3^+^ cells. These cells had increased Wnt/β-catenin signaling, and activation of this pathway resulted in M2 macrophage infiltration via the Cxcl14 chemokine. This resulted in fibrosis. The authors reported that their findings suggest that the Cxcl14 stromal cell and macrophage interaction is a promising target for the treatment of fibrosis mediated by Wnt/β-catenin signaling. Given the previous studies around the association of TPPP3 with β-catenin and downstream pathways, this interaction may also be a viable therapeutic target [25,52].

## 5. Endothelial Dysfunction

Palmitic acid is one of the most common free fatty acids in the body [74] and is known to induce lipotoxicity, a primary cause of endothelial dysfunction [75]. Using human umbilical vein endothelial cells, Liu et al. found that in the presence of palmitic acid, TPPP3 expression significantly increased [76]. Additionally, reactive oxygen species (ROS) formation was significantly increased and led to cell death and decreased tube formation. This was mediated by TPPP3 interacting with, and stabilizing, voltage-dependent anion channel 1 (VDAC1). VDAC1 is the most abundant anion channel in the mitochondrial membrane and is responsible for the transport of metabolites in and out of the mitochondria, including ROS [77].

Interestingly, they also found that TPPP3 promotes ROS formation and through mass spectrometry, found it has an interaction with VDAC1. While this leads to damage and apoptosis in endothelial cells, it is conceivable that this could also be cancer promoting, as adequate ROS levels are important for cancer proliferation, differentiation and metastasis [78]. However, similar to the findings in endothelial cells, high levels of ROS ultimately led to cellular damage and cell death.

## 6. Neurodegenerative Diseases

TPPP3 has also been explored in the context of neuron regeneration and neurodegenerative diseases. A study in zebrafish found that *tppp3* mRNA is a target of miR-133b in the regulation of axonal regeneration [79]. Further studies identified Tppp3 as a marker of retinal ganglion cells and that Tppp3 overexpression promotes retinal ganglion cell regeneration and neurite outgrowth [80]. Despite its name, tubulin polymerization promoting protein, there is surprisingly little research associating TPPP3 with tubulin or microtubules. Although this has not been experimentally validated, the role of TPPP3 in neuron regeneration is potentially due to its abilities to bundle and bind to microtubules, since microtubule bundling is extremely important for neuron structure [81]. TPPP3 has also been investigated in the context of neurodegenerative disease, specifically in the context of alpha-synucleinopathies [82]. Alpha-synucleinopathies are a group of neurodegenerative diseases characterized by aggregates of hyperphosphorylated α-synuclein that lead to neuron death [83]. Some of these diseases include Parkinson’s disease, multiple system atrophy, and diffuse Lewy body disease. In 2004, TPPP1 was identified as a common marker for alpha-synucleinopathies [11]. The researchers found that TPPP1 and α-synuclein were co-enriched and co-localized in patients with Parkinson’s disease and multiple system atrophy. However, they also found that TPPP3 will dimerize with itself and TPPP1 but had little binding to α-synuclein and therefore did little in the promotion of α-synuclein aggregation, like TPPP1 [82]. Although the strong affinity to TPPP1 resulted in the destruction of the TPPP1- α-synuclein complexes that promote these diseases, TPPP3 may have an inhibitory effect in alpha-synucleinopathies.

Together, these studies indicate that TPPP3 may play a significant role in neuron biology and neurodegenerative disease and further research is needed to further characterize its roles in these cell types.

## 7. Other Studies

Beyond these broad categories, there have been a number of sequencing studies that have found significant changes in TPPP3 gene expression [84,85,86,87,88,89,90,91] (Table 2). While TPPP3 is not the focus of these studies, these large datasets are extremely valuable as they allow for future hypothesis generation and inform the direction of new studies. 

## 8. Conclusions

Several studies have supported the role of TPPP3 in promoting proliferation and metastasis. However, TPPP3 may operate through distinct, cancer-type-specific pathways. TPPP3 modulates STAT3/Twist1 signaling in non-small-cell lung carcinoma and colorectal cancer, promoting proliferation, migration and invasion [18,22,24]. In breast cancer, loss of TPPP3 is also associated with decreased proliferation, migration and invasion. However, it is operating on the NF-kB/COX2 axis, a commonly altered pathway in breast cancer [33]. Loss of TPPP3 in endometrial cancer also resulted in decreased proliferation, migration, and invasion; however, a mechanism was not proposed. However, they did identify TPPP3 as a target of microRNA-1827 which has the potential to be leveraged as a therapeutic option in cancers with aberrant TPPP3 expression [32,92]. In glioblastoma, TPPP3 affects the Snail pathway, a common marker of EMT [39]. Snail and Twist are both promoters of EMT and can even modulate each other’s expression [40]. However, even though TPPP3 has been shown to be involved in both pathways, Twist1 in non-small-cell lung carcinoma and Snail1 in glioblastoma, there is no evidence that the expression of the other is modulated in these cancers. In hepatocellular carcinoma, TPPP3 is involved in a more structural context. TPPP3 allowed hepatocellular carcinoma cells to withstand fluid shear stress better than TPPP3 knockdown cells, making them more viable during metastasis [42]. Further studies are needed to elucidate the role of TPPP3 in enabling cells to withstand fluid shear stress.

Conversely, there are certain cancers where TPPP3 is associated with better survival. There are multiple head and neck cancers where tumor samples had lower TPPP3 expression than the surrounding area and was correlated with worse survival [45,46]. Interestingly, elevated levels of TPPP3 expression were associated with certain immune cell infiltration into the tumor. Pancreatic ductal adenocarcinoma tumors also showed worse overall survival with lower TPPP3 expression [43]. Further studies will be needed to understand the protective effects of TPPP3 in these tumors.

Even with all this research assessing the impact of TPPP3 on these different cancers, the question remains—what does TPPP3 do? It is involved in multiple pathways, but the actual role it plays in proliferation and metastasis in many of these cancers remains unknown. It can bundle microtubules, but it likely has many other binding partners that facilitate its role in tumorigenesis and metastasis.

However, in early pregnancy, it does seem to have a direct role in the β-catenin/NF-kB/Cox2 pathway [25,52]. TPPP3 is able to be pulled down with β-catenin, indicating that it is a binding partner, or at the very least binds to a complex that β-catenin binds to. Loss of TPPP3 decreases β-catenin signaling and results in embryo implantation failure and endometrial decidualization failure. This could potentially inform research about the role of TPPP3 in cancer, given it is already associated with NF-kB/COX2 pathways in breast cancer [33]. Investigating how it interacts with the β-catenin pathway in cancer may potentially lead to an understanding of its role in cancer proliferation and invasion.

Consistent with its seemingly broad functions, it is also a marker of certain stem cell populations; specifically, Tppp3+ tendon stem cells are associated with wound healing/regeneration and fibrosis [64,66,70]. The exact role of Tppp3 is still unknown. TPPP3 is not ubiquitously expressed throughout the body, so it must have unique functions as to be expressed in a specific tendon stem cell population.

We can further assess its functions through its physical interaction with VDAC1. TPPP3 can stabilize this channel to allow for the release of ROS in the presence of palmitic acid [76]. This is a very different function from its prior contexts in cancer, early pregnancy, and tendon injury. However, it provides further insight into the binding partners of TPPP3, furthering our understanding of it in normal physiology.

## 9. Future Directions

Despite the advances in our understanding of TPPP3, there still remain large gaps that need to be filled. One such gap is the discrepancy seen in the role TPPP3 plays in cancer, whether it be oncogenic or tumor suppressive. More studies will need to be conducted to determine how TPPP3 knockdown or overexpression affects proliferation, migration, and invasion. Additionally, it could potentially inform us on certain cell type specificities of different TPPP3 functions.

We also do not understand the regulatory mechanisms controlling TPPP3 expression. No prior research to our knowledge has been done uncovering the transcription factors or epigenetic regulation involved in TPPP3 expression. This would be valuable in not only further characterizing TPPP3 in health and disease, but it would also help in the development of more targeted therapies for these diseases, particularly in cancer.

Targeting TPPP3 in disease is also a problem. There are currently no drugs or inhibitors that can target it. There has been one study that found PI3K inhibitor BKM-120, also called buparlisib, could potentially inhibit TPPP3 expression, although this was only seen in sequencing data and not experimentally validated [93]. However, given that TPPP3 has been shown to be involved in PI3K/AKT signaling, this could be a viable therapeutic avenue to explore. We also know there are miRNAs that target TPPP3 [32,37] and there are methods of delivering miRNAs systemically to different tissues with therapeutic effect [94,95]. However, off-target toxicities and delivery difficulties, among other things, still remain a large challenge [96].

Lastly, one of the largest gaps in our knowledge of TPPP3 is identifying its interaction partners and how it exerts its effects. In cancer, we know that it is involved in proliferation and metastasis, because silencing of TPPP3 results in a decrease in expression of proliferation and metastasis markers, followed by a likewise decrease in proliferative and metastatic potential. However, many of its interaction partners remain elusive. In the case of embryo implantation and decidualization, it was found to interact with β-catenin, or at least a complex with β-catenin, but we do not know how the loss of its expression directly results in implantation failure and decidualization failure. Likewise in the musculoskeletal context, we know TPPP3 acts as a marker of specific stem cell populations, but what its role is intracellularly is still unknown. These future studies will provide insights into the role of TPPP3 in human health and disease.

## Figures and Tables

**Table 1:** Cancers where TPPP3 has been implicated, the cell lines used in the study, whether TPPP3 was found to be protective or tumorigenic, and pathways TPPP3 was implicated in.

Cell Type	Cell Line	Role of TPPP3	Altered Pathways	Ref
Cervical cancer	HeLa	**Tumorigenic** Proliferation Mitosis		[14]
Lewis lung carcinoma		**Tumorigenic** Proliferation Mitosis Suppression induces apoptosis		[17]
Non-small cell lung cancer	A549 H1229	**Tumorigenic** Proliferation Suppression induces apoptosis	STAT3/AKT	[18]
Non-small cell lung cancer	95-C SPC-A1 A549 H1229 Patient Samples (treatment naive)	**Tumorigenic** Proliferation Invasion Migration Metastasis Suppression induces apoptosis	STAT3/Twist1	[22]
Colorectal cancer	SW480 HT29 RKO LOVO SW620 HUVEC	**Tumorigenic** Proliferation Invasion Migration Metastasis Angiogenesis	STAT3 VEGF/P21	[24]
Breast cancer	MCF-7 T47D Patient samples (treatment naïve)	**Tumorigenic** Proliferation Invasion Migration	NF-kB/COX2	[33]
Endometrial cancer	Ishikawa KLE RL-95 AN3CA Patient Samples (treatment naïve)	**Tumorigenic** Proliferation Invasion Migration TPPP3 is targeted by mIR-1827		[32]
Glioblastoma	U87ZMG U118 A172 LN229 U251	**Tumorigenic** Proliferation Invasion Migration EMT Suppression induces apoptosis	SNAIL1	[39]
Hepatocellular carcinoma	HepG2 SK-Hep-1	**Tumorigenic** Metastasis		[22]
Pancreatic ductal adenocarcinoma	FFPE patient samples	**Protective** High expression associated with longer survival.		[43]
Nasopharyngeal carcinoma	Patient samples	**Protective** Lower TPPP3 expression in samples compared to normal tissue. Higher expression associated with better survival	Immune cell infiltration into tumors	[44]
Oral squamous cell carcinoma	Patient samples	**Protective** Lower TPPP3 expression in samples compared to normal tissue. Higher expression associated with better survival	Immune cell infiltration into tumors	[45]

**Table 2:** Other sequencing and proteomic studies that found TPPP3 as highly differentially expressed.

Disease/Context	Data Type	Regulation	Ref
Diabetic Retinopathy	Proteome-wide associate studies		[84]
Epilepsy	RNA-seq iTRAQ proteomics	Downregulated in patients with epilepsy	[85]
Lung cancer and COPD	Proteomics	Upregulated in COPD, lung cancer, and COPD and lung cancer groups	[86]
High blood pressure	microarray	Upregulated in monocytes associated with increased blood pressure.	[87]
Ulcerative colitis	RNA-seq	Downregulated in ulcerative colitis group compared to control	[88]
Non-ulcerative bladder pain syndrome	RNA-seq	Biomarker for non-ulcerative bladder pain syndrome	[89]
Sarcopenia	RNA-seq	Upregulated in patients with sarcopenia	[90]
Alveolar regeneration	RNA-seq	Upregulated during AT2 cell differentiation. TPPP3 was reduced in Tert^−\−^ mice.	[91]

## Data Availability

No new data were created or analyzed in this study. Data sharing is not applicable to this article.
